# Analysis and interpretation of dynamic FDG PET oncological studies using data reduction techniques

**DOI:** 10.1186/1475-925X-6-36

**Published:** 2007-10-03

**Authors:** Sotiris Pavlopoulos, Trias Thireou, George Kontaxakis, Andres Santos

**Affiliations:** 1 Biomedical Engineering Laboratory, School of Electrical and Computer Engineering, National Technical University of Athens, GR-15773 Athens, Greece; 2 Dpto. de Ingeniería Electrónica, ETSI Telecomunicación, Universidad Politécnica de Madrid, Madrid, Spain

## Abstract

**Background:**

Dynamic positron emission tomography studies produce a large amount of image data, from which clinically useful parametric information can be extracted using tracer kinetic methods. Data reduction methods can facilitate the initial interpretation and visual analysis of these large image sequences and at the same time can preserve important information and allow for basic feature characterization.

**Methods:**

We have applied principal component analysis to provide high-contrast parametric image sets of lower dimensions than the original data set separating structures based on their kinetic characteristics. Our method has the potential to constitute an alternative quantification method, independent of any kinetic model, and is particularly useful when the retrieval of the arterial input function is complicated. In independent component analysis images, structures that have different kinetic characteristics are assigned opposite values, and are readily discriminated. Furthermore, novel similarity mapping techniques are proposed, which can summarize in a single image the temporal properties of the entire image sequence according to a reference region.

**Results:**

Using our new cubed sum coefficient similarity measure, we have shown that structures with similar time activity curves can be identified, thus facilitating the detection of lesions that are not easily discriminated using the conventional method employing standardized uptake values.

## Background

In oncology, Positron Emission Tomography (PET) studies are routinely used for tumor diagnosis, detection of metastases, and treatment evaluation. Dynamic PET (i.e., temporal sequences of images at the same bed position) offers differential diagnostic information, and therefore represents an accurate approach to quantifying radiotracer kinetics However, the quantitative analysis of dynamic PET sequences often requires complex analysis using compartmental [1,2] or non-compartmental models [3], where many difficulties must be overcome, such as determination of the input function of the concentration of the radioactive tracer in the plasma [4], the intrinsic inaccuracies at the time of selecting the appropriate compartmental model [5], or carrying out time-consuming computations involving a large volume of image data that has to be processed [6].

In this work, we investigated the use of principal component analysis (PCA) [7,8], independent component analysis (ICA) [9,10], and similarity mapping (SM) [11] techniques to reduce the initial volume of image data to a smaller and more comprehensive and easily managed set of parametric images. Blind source separation methods, such as PCA and ICA, can provide component extraction and time courses in dynamic PET studies without requiring any explicit knowledge of the system transfer function which is needed in the case of image reconstruction. Furthermore, such methods have the advantage in producing results in very short time, as they have little computational complexity, and can provide an accurate tool for the support of both visual inspections and the subsequent detailed kinetic analysis of the dynamic series using compartmental or non-compartmental models. As an alternative data reduction technique, SM permits the extraction of information from a sequence of images on the physiological behavior of the system under study, which is not revealed by visual inspection of the image sets.

In the following subsections, the above techniques are presented in more detail.

### A. Principal Component Analysis

PCA explains the variance-covariance of a set of variables using a few linear combinations of the data to achieve data reduction and thus facilitates data interpretation [12]. Although N components are required to reproduce the total system variability, often much of this variability can be accounted for by a small number, p, of the principal components, which can be considered as containing the same information as the original data set (excluding the contribution of noise, which can be attributed to the remaining data). These components can then replace the initial N variables, and the original data set, consisting of k measurements of N variables, is reduced to a data set consisting of k measurements of p principal components.

PCA has been early adopted in the applied sciences [13], with the main goal of investigating if the first few principal components account for most of the variation in the original data [14]. The same methodology has been applied in the field of medical imaging, particularly in functional magnetic resonance imaging (fMRI) [15], and in nuclear medicine, where this type of analysis has been employed as a tool for denoising dynamic image sequences [16,17].

In dynamic PET images, the first few principal components (PC) constitute a reduced set of the principal component images (PCI) that can be considered as representing a "summary" of the kinetic information that is contained in the original study frames [18], and can therefore be used to extract basic information for initial evaluations in dynamic studies in oncological applications [19,20], as well as in neurological studies, where PCA is particularly useful in the follow-up of Parkinson's disease patients [21,22].

Furthermore, PCA techniques have been proposed and applied in dynamic PET [17] as a filtering method in the time domain with the reconstruction being performed component by component in the PC (Karhunen-Loewe, KL) domain, followed by a recovery of the spatial distribution of the radioactivity in the source using an inverse KL transform [23]. This sinogram-domain PCA (S-PCA) for dynamic PET image reconstruction has been improved by researchers on using noise normalization and optimal sampling techniques [24], and in regard to the resulting data sets, a segmentation method has been recently proposed [25] that could extract noninvasively the input function (arterial time-activity curve) in the kinetic analysis of a dynamic study.

### B. Independent Component Analysis

ICA [26] is another data-driven statistical technique that can be used for blind separation of sources, and has early found application in medical signals [27] and image analysis [28]. The observed data are assumed to be an unknown linear mixture of unobserved independent source signals, which can be recovered with no prior information or other knowledge of the system response function.

ICA has been recently shown to produce promising results in the analysis of task-related fMRI techniques [29], as well as in the extraction of the input function [30] and the separation of functional components in gated myocardial PET studies [31]. ICA has also been recently applied to extract the plasma time activity curve (TAC) in dynamic FDG PET brain studies [32].

Spatial ICA (sICA) [33] can be used to decompose an image sequence into a set of mutually independent component (IC) source images and a corresponding set of unconstrained time courses, based on the assumption that the probability density function (PDF) of the independent sources is highly kurtotic and symmetric. Since this assumption is not necessary for dynamic PET data sets, skew-sICA [34] has been applied to dynamic PET data sets using the code developed by Stone et al. [29]. Skew-sICA assumes that images are characterized by the skewness (rather than the peakedness) of their PDFs, which is consistent with spatially localized regions of activity.

Singular value decomposition (SVD) is performed to decorrelate input images, and the eigenvalues (*λ*) are normalized such that:

Σ*λ *= number of frames

The eigenvectors with the largest variance and eigenvalues greater than unity are selected as the ICA input data, and the remaining noise components are discarded.

### C. Similarity Mapping

As mentioned earlier, the main goal of the initial evaluation step in dynamic oncological PET studies is to assess the accuracy in localizing and staging primary tumors and metastases. SM methods create a temporal match of the intensity values of the pixels in the image sequence with the pixels from a selected reference region of interest (rROI). Therefore, SM segments multidimensional images into regions according to their temporal properties rather than their spatial properties, which makes it useful for the temporal analysis of dynamic PET series.

In medical imaging, the application of SM to CT images of rabbits with focal cerebral ischemia allowed for the identification of small differences in the temporal kinetics around the infarct [35]. In an analysis of dynamic scintigraphic cardiac images, SM applied to regions of similar temporal behavior (i.e., covariance images) captured the essential elements of the sequence while reducing the amount of image data presented to the clinician for diagnostic interpretation [36]. The similarity measures applied in dynamic MRI studies [37,38] are based on the calculation of the correlation (COR) and the normalized correlation (NCOR) coefficients. However, for low contrast PET images, as discussed in the Results section, these similarity coefficients are inadequate, and therefore for the purposes of our study we have introduced additional similarity measures as described in the Methods section below.

## Methods

We have used PCA, ICA and SM techniques and applied them to dynamic 2-deoxy-2 [18F]fluoro-D-glucose (18F-FDG) PET studies, first to realistic synthetic data sets, and then to clinical data from oncological patients.

In order to improve the performance of PCAs for dynamic PET we decided to introduce data preprocessing. From the several data preprocessing methods described in the literature [39], we have selected and used preprocessing by the column sum (PCS), where the data are divided columnwise using the column sum:

zij=yij(∑i=1myij)−1
 MathType@MTEF@5@5@+=feaafiart1ev1aaatCvAUfKttLearuWrP9MDH5MBPbIqV92AaeXatLxBI9gBaebbnrfifHhDYfgasaacH8akY=wiFfYdH8Gipec8Eeeu0xXdbba9frFj0=OqFfea0dXdd9vqai=hGuQ8kuc9pgc9s8qqaq=dirpe0xb9q8qiLsFr0=vr0=vr0dc8meaabaqaciaacaGaaeqabaqabeGadaaakeaacqWG6bGEdaWgaaWcbaGaemyAaKMaemOAaOgabeaakiabg2da9iabdMha5naaBaaaleaacqWGPbqAcqWGQbGAaeqaaOGaeiikaGYaaabCaeaacqWG5bqEdaWgaaWcbaGaemyAaKMaemOAaOgabeaaaeaacqWGPbqAcqGH9aqpcqaIXaqmaeaacqWGTbqBa0GaeyyeIuoakiabcMcaPmaaCaaaleqabaGaeyOeI0IaeGymaedaaaaa@4596@

where y_ij _and z_ij _are the original and the final value of pixel i (i = 1,..., m) of frame j (j = 1,..., n), respectively.

Similarly, and in order to avoid the known problem of overfitting [26] in ICA, the PCS preprocessing method has been also applied to image data before the application of ICA.

Regarding SM, and in order to overcome the limitations of COR and NCOR coefficients when applied to low contrast PET images, we introduced additional similarity measures of: (i) the sum of squares (SSQ); (ii) the sum of cubes (SC); (iii) the squared sum (SQS); and (iv) the cubed sum (CS) coefficients:

CORij=∑n=1NVijnRn∑n=1NVijn2∑n=1NRn2
 MathType@MTEF@5@5@+=feaafiart1ev1aaatCvAUfKttLearuWrP9MDH5MBPbIqV92AaeXatLxBI9gBaebbnrfifHhDYfgasaacH8akY=wiFfYdH8Gipec8Eeeu0xXdbba9frFj0=OqFfea0dXdd9vqai=hGuQ8kuc9pgc9s8qqaq=dirpe0xb9q8qiLsFr0=vr0=vr0dc8meaabaqaciaacaGaaeqabaqabeGadaaakeaacqWGdbWqcqWGpbWtcqWGsbGudaWgaaWcbaGaemyAaKMaemOAaOgabeaakiabg2da9maalaaabaWaaabCaeaacqWGwbGvdaWgaaWcbaGaemyAaKMaemOAaOMaemOBa4gabeaakiabdkfasnaaBaaaleaacqWGUbGBaeqaaaqaaiabd6gaUjabg2da9iabigdaXaqaaiabd6eaobqdcqGHris5aaGcbaWaaOaaaeaadaaeWbqaaiabdAfawnaaDaaaleaacqWGPbqAcqWGQbGAcqWGUbGBaeaacqaIYaGmaaaabaGaemOBa4Maeyypa0JaeGymaedabaGaemOta4eaniabggHiLdGcdaaeWbqaaiabdkfasnaaDaaaleaacqWGUbGBaeaacqaIYaGmaaaabaGaemOBa4Maeyypa0JaeGymaedabaGaemOta4eaniabggHiLdaaleqaaaaaaaa@5ACF@

NCORij=∑n=1N(Vijn−μVij)(Rn−μR)∑n=1N(Vijn−μVij)2∑n=1N(Rn−μR)2
 MathType@MTEF@5@5@+=feaafiart1ev1aaatCvAUfKttLearuWrP9MDH5MBPbIqV92AaeXatLxBI9gBaebbnrfifHhDYfgasaacH8akY=wiFfYdH8Gipec8Eeeu0xXdbba9frFj0=OqFfea0dXdd9vqai=hGuQ8kuc9pgc9s8qqaq=dirpe0xb9q8qiLsFr0=vr0=vr0dc8meaabaqaciaacaGaaeqabaqabeGadaaakeaacqWGobGtcqWGdbWqcqWGpbWtcqWGsbGudaWgaaWcbaGaemyAaKMaemOAaOgabeaajaaqcqGH9aqpkmaalaaajaaqbaGcdaaeWbqaaiabcIcaOiabdAfawnaaBaaaleaacqWGPbqAcqWGQbGAcqWGUbGBaeqaaOGaeyOeI0ccciGae8hVd02aaSbaaSqaaiabdAfawjabdMgaPjabdQgaQbqabaGccqGGPaqkcqGGOaakcqWGsbGudaWgaaWcbaGaemOBa4gabeaakiabgkHiTiab=X7aTnaaBaaaleaacqWGsbGuaeqaaOGaeiykaKcaleaacqWGUbGBcqGH9aqpcqaIXaqmaeaacqWGobGta0GaeyyeIuoaaKaaafaakmaakaaajaaqbaGcdaaeWbqaaiabcIcaOiabdAfawnaaBaaaleaacqWGPbqAcqWGQbGAcqWGUbGBaeqaaOGaeyOeI0Iae8hVd02aaSbaaSqaaiabdAfawjabdMgaPjabdQgaQbqabaGccqGGPaqkdaahaaWcbeqaaiabikdaYaaaaeaacqWGUbGBcqGH9aqpcqaIXaqmaeaacqWGobGta0GaeyyeIuoakmaaqahabaGaeiikaGIaemOuai1aaSbaaSqaaiabd6gaUbqabaGccqGHsislcqWF8oqBdaWgaaWcbaGaemOuaifabeaakiabcMcaPmaaCaaaleqabaGaeGOmaidaaaqaaiabd6gaUjabg2da9iabigdaXaqaaiabd6eaobqdcqGHris5aaqcbauabaaaaaaa@7A44@

SSQij=∑n=1N(Vijn−μVij)2(Rn−μR)2μR∑n=1N(Vijn−μVij)2∑n=1N(Rn−μR)2
 MathType@MTEF@5@5@+=feaafiart1ev1aaatCvAUfKttLearuWrP9MDH5MBPbIqV92AaeXatLxBI9gBaebbnrfifHhDYfgasaacH8akY=wiFfYdH8Gipec8Eeeu0xXdbba9frFj0=OqFfea0dXdd9vqai=hGuQ8kuc9pgc9s8qqaq=dirpe0xb9q8qiLsFr0=vr0=vr0dc8meaabaqaciaacaGaaeqabaqabeGadaaakeaacqWGtbWucqWGtbWucqWGrbqudaWgaaWcbaGaemyAaKMaemOAaOgabeaajaaqcqGH9aqpkmaalaaajaaqbaGcdaaeWbqaaiabcIcaOiabdAfawnaaBaaaleaacqWGPbqAcqWGQbGAcqWGUbGBaeqaaOGaeyOeI0ccciGae8hVd02aaSbaaSqaaiabdAfawjabdMgaPjabdQgaQbqabaGccqGGPaqkdaahaaWcbeqaaiabikdaYaaakiabcIcaOiabdkfasnaaBaaaleaacqWGUbGBaeqaaOGaeyOeI0Iae8hVd02aaSbaaSqaaiabdkfasbqabaGccqGGPaqkdaahaaWcbeqaaiabikdaYaaaaeaacqWGUbGBcqGH9aqpcqaIXaqmaeaacqWGobGta0GaeyyeIuoaaKaaafaacqWF8oqBkmaaBaaaleaacqWGsbGuaeqaaOWaaOaaaeaadaaeWbqaaiabcIcaOiabdAfawnaaBaaaleaacqWGPbqAcqWGQbGAcqWGUbGBaeqaaOGaeyOeI0Iae8hVd02aaSbaaSqaaiabdAfawjabdMgaPjabdQgaQbqabaGccqGGPaqkdaahaaWcbeqaaiabikdaYaaakmaaqahabaGaeiikaGIaemOuai1aaSbaaSqaaiabd6gaUbqabaGccqGHsislcqWF8oqBdaWgaaWcbaGaemOuaifabeaakiabcMcaPmaaCaaaleqabaGaeGOmaidaaaqaaiabd6gaUjabg2da9iabigdaXaqaaiabd6eaobqdcqGHris5aaWcbaGaemOBa4Maeyypa0JaeGymaedabaGaemOta4eaniabggHiLdaaleqaaaaaaaa@7E0F@

SCij=∑n=1N(Vijn−μVij)3(Rn−μR)3μR2∑n=1N(Vijn−μVij)2∑n=1N(Rn−μR)2
 MathType@MTEF@5@5@+=feaafiart1ev1aaatCvAUfKttLearuWrP9MDH5MBPbIqV92AaeXatLxBI9gBaebbnrfifHhDYfgasaacH8akY=wiFfYdH8Gipec8Eeeu0xXdbba9frFj0=OqFfea0dXdd9vqai=hGuQ8kuc9pgc9s8qqaq=dirpe0xb9q8qiLsFr0=vr0=vr0dc8meaabaqaciaacaGaaeqabaqabeGadaaakeaacqWGtbWucqWGdbWqdaWgaaWcbaGaemyAaKMaemOAaOgabeaajaaqcqGH9aqpkmaalaaajaaqbaGcdaaeWbqaaiabcIcaOiabdAfawnaaBaaaleaacqWGPbqAcqWGQbGAcqWGUbGBaeqaaOGaeyOeI0ccciGae8hVd02aaSbaaSqaaiabdAfawjabdMgaPjabdQgaQbqabaGccqGGPaqkdaahaaWcbeqaaiabiodaZaaakiabcIcaOiabdkfasnaaBaaaleaacqWGUbGBaeqaaOGaeyOeI0Iae8hVd02aaSbaaSqaaiabdkfasbqabaGccqGGPaqkdaahaaWcbeqaaiabiodaZaaaaeaacqWGUbGBcqGH9aqpcqaIXaqmaeaacqWGobGta0GaeyyeIuoaaKaaafaacqWF8oqBkmaaDaaaleaaieGacqGFsbGuaeaaieaacqqFYaGmaaGcdaGcaaqaamaaqahabaGaeiikaGIaemOvay1aaSbaaSqaaiabdMgaPjabdQgaQjabd6gaUbqabaGccqGHsislcqWF8oqBdaWgaaWcbaGaemOvayLaemyAaKMaemOAaOgabeaakiabcMcaPmaaCaaaleqabaGaeGOmaidaaOWaaabCaeaacqGGOaakcqWGsbGudaWgaaWcbaGaemOBa4gabeaakiabgkHiTiab=X7aTnaaBaaaleaacqWGsbGuaeqaaOGaeiykaKYaaWbaaSqabeaacqaIYaGmaaaabaGaemOBa4Maeyypa0JaeGymaedabaGaemOta4eaniabggHiLdaaleaacqWGUbGBcqGH9aqpcqaIXaqmaeaacqWGobGta0GaeyyeIuoaaSqabaaaaaaa@7DBF@

SQSij=(∑n=1N(Vijn−μVij)(Rn−μR))2μR∑n=1N(Vijn−μVij)2∑n=1N(Rn−μR)2
 MathType@MTEF@5@5@+=feaafiart1ev1aaatCvAUfKttLearuWrP9MDH5MBPbIqV92AaeXatLxBI9gBaebbnrfifHhDYfgasaacH8akY=wiFfYdH8Gipec8Eeeu0xXdbba9frFj0=OqFfea0dXdd9vqai=hGuQ8kuc9pgc9s8qqaq=dirpe0xb9q8qiLsFr0=vr0=vr0dc8meaabaqaciaacaGaaeqabaqabeGadaaakeaacqWGtbWucqWGrbqucqWGtbWudaWgaaWcbaGaemyAaKMaemOAaOgabeaajaaqcqGH9aqpkmaalaaajaaqbaGccqGGOaakdaaeWbqaaiabcIcaOiabdAfawnaaBaaaleaacqWGPbqAcqWGQbGAcqWGUbGBaeqaaOGaeyOeI0ccciGae8hVd02aaSbaaSqaaiabdAfawjabdMgaPjabdQgaQbqabaGccqGGPaqkcqGGOaakcqWGsbGudaWgaaWcbaGaemOBa4gabeaakiabgkHiTiab=X7aTnaaBaaaleaacqWGsbGuaeqaaOGaeiykaKIaeiykaKYaaWbaaSqabeaacqaIYaGmaaaabaGaemOBa4Maeyypa0JaeGymaedabaGaemOta4eaniabggHiLdaajaaqbaGae8hVd0McdaWgaaWcbaGaemOuaifabeaakmaakaaabaWaaabCaeaacqGGOaakcqWGwbGvdaWgaaWcbaGaemyAaKMaemOAaOMaemOBa4gabeaakiabgkHiTiab=X7aTnaaBaaaleaacqWGwbGvcqWGPbqAcqWGQbGAaeqaaOGaeiykaKYaaWbaaSqabeaacqaIYaGmaaGcdaaeWbqaaiabcIcaOiabdkfasnaaBaaaleaacqWGUbGBaeqaaOGaeyOeI0Iae8hVd02aaSbaaSqaaiabdkfasbqabaGccqGGPaqkdaahaaWcbeqaaiabikdaYaaaaeaacqWGUbGBcqGH9aqpcqaIXaqmaeaacqWGobGta0GaeyyeIuoaaSqaaiabd6gaUjabg2da9iabigdaXaqaaiabd6eaobqdcqGHris5aaWcbeaaaaaaaa@7E98@

CSij=(∑n=1N(Vijn−μVij)(Rn−μR))3μR2∑n=1N(Vijn−μVij)2∑n=1N(Rn−μR)2
 MathType@MTEF@5@5@+=feaafiart1ev1aaatCvAUfKttLearuWrP9MDH5MBPbIqV92AaeXatLxBI9gBaebbnrfifHhDYfgasaacH8akY=wiFfYdH8Gipec8Eeeu0xXdbba9frFj0=OqFfea0dXdd9vqai=hGuQ8kuc9pgc9s8qqaq=dirpe0xb9q8qiLsFr0=vr0=vr0dc8meaabaqaciaacaGaaeqabaqabeGadaaakeaacqWGdbWqcqWGtbWudaWgaaWcbaGaemyAaKMaemOAaOgabeaajaaqcqGH9aqpkmaalaaajaaqbaGccqGGOaakdaaeWbqaaiabcIcaOiabdAfawnaaBaaaleaacqWGPbqAcqWGQbGAcqWGUbGBaeqaaOGaeyOeI0ccciGae8hVd02aaSbaaSqaaiabdAfawjabdMgaPjabdQgaQbqabaGccqGGPaqkcqGGOaakcqWGsbGudaWgaaWcbaGaemOBa4gabeaakiabgkHiTiab=X7aTnaaBaaaleaacqWGsbGuaeqaaOGaeiykaKIaeiykaKYaaWbaaSqabeaacqaIZaWmaaaabaGaemOBa4Maeyypa0JaeGymaedabaGaemOta4eaniabggHiLdaajaaqbaGae8hVd0McdaqhaaWcbaGaemOuaifabaGaeGOmaidaaOWaaOaaaeaadaaeWbqaaiabcIcaOiabdAfawnaaBaaaleaacqWGPbqAcqWGQbGAcqWGUbGBaeqaaOGaeyOeI0Iae8hVd02aaSbaaSqaaiabdAfawjabdMgaPjabdQgaQbqabaGccqGGPaqkdaahaaWcbeqaaiabikdaYaaakmaaqahabaGaeiikaGIaemOuai1aaSbaaSqaaiabd6gaUbqabaGccqGHsislcqWF8oqBdaWgaaWcbaGaemOuaifabeaakiabcMcaPmaaCaaaleqabaGaeGOmaidaaaqaaiabd6gaUjabg2da9iabigdaXaqaaiabd6eaobqdcqGHris5aaWcbaGaemOBa4Maeyypa0JaeGymaedabaGaemOta4eaniabggHiLdaaleqaaaaaaaa@7E42@

where N is the frame number, V_ijn _is the value of pixel (i, j) in frame n, R_n _is the value of the TAC and *μ*_R _is the mean value of the TAC in the rROI, respectively, and *μ*_Vij _is the mean value of the TAC of pixel (i, j).

The application of SM to a dynamic PET study results in one map per slice, where each pixel value represents the degree of temporal similarity of the selected region to the reference region. Both the COR and the NCOR measures are normalized for proportional differences, while only the NCOR data are normalized for additive differences, and therefore, TACs that differ by an additive constant cannot be distinguished using NCOR, as they can in the case of COR [37]. The SSQ and SQS measures provide a similarity measure that is normalized for additive differences and negative values, whereas the SC and CS measures are normalized for additive differences.

### A. Simulated Data

**Figure 1:**
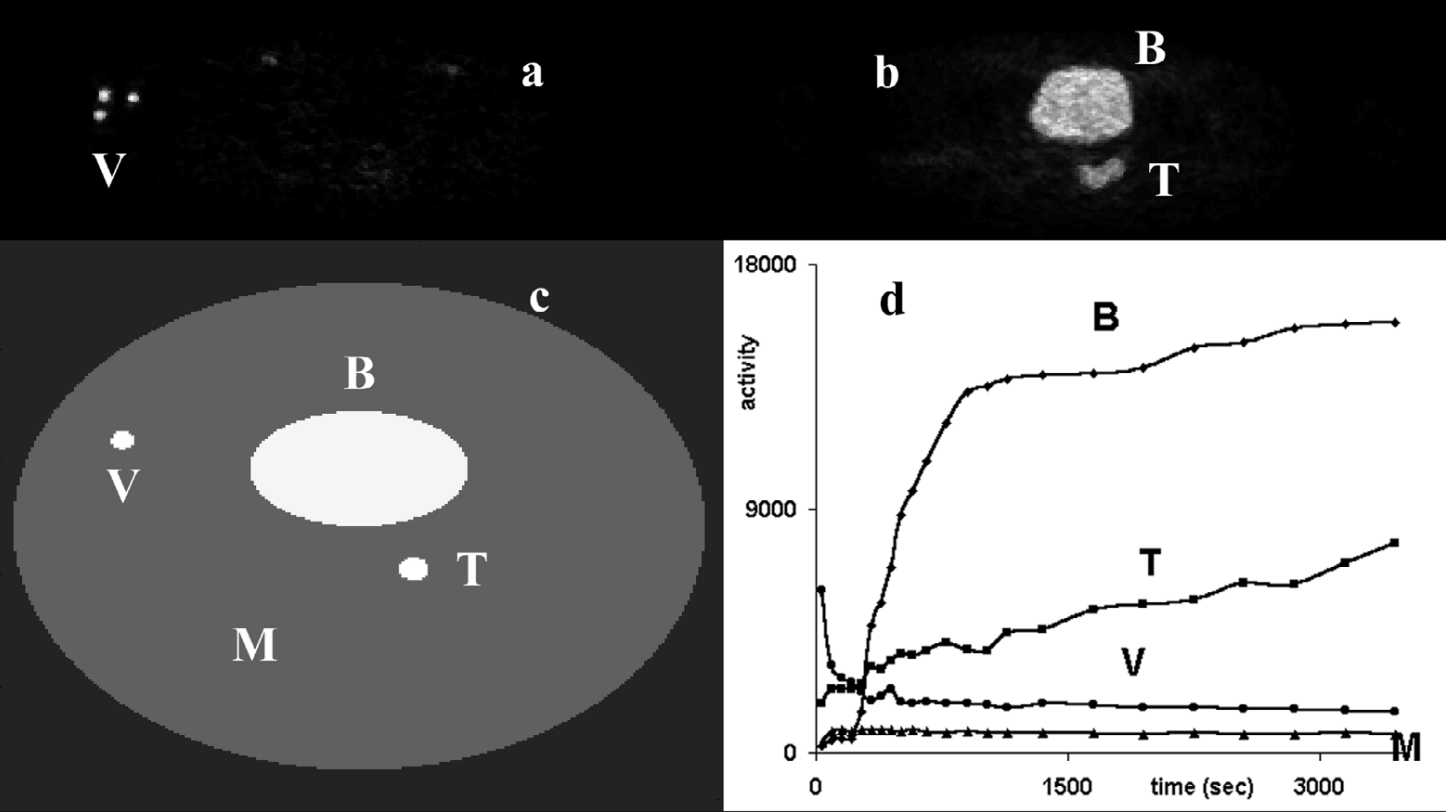
Two frames (a) and (b) from a real dynamic PET study used for the formation of a simulated dynamic PET phantom image series, (c). The TACs (d) from the study were used for the definition of the corresponding TAC functions of the phantom. The phantom consisted of a large ellipse (M) corresponding to the normal tissue mass and three smaller ellipses corresponding to the bladder (B), tumor (T), and a blood vessel (V).

We applied the data reduction techniques to synthetic dynamic data from a digital phantom (figure 1), which simulated a single-slice image series from an 18F-FDG PET study of a colorectal tumor recurrence.

The phantom image consisted of a large ellipse (M) corresponding to the normal tissue masses (which, in real PET scans apart from muscle may include gut, fat, fine vasculature, other soft tissue structures, and bones of the pelvis), and three smaller ellipses corresponding to the bladder (B), tumor (T), and blood vessel (V).

The TACs were derived from ROIs placed over the structures of a real, clinical dynamic 18F-FDG PET study, including the noise characteristics of the measured data. The acquisition and image reconstruction protocols used were those described in the next subsection.

### B. Clinical Data

**Figure 2:**
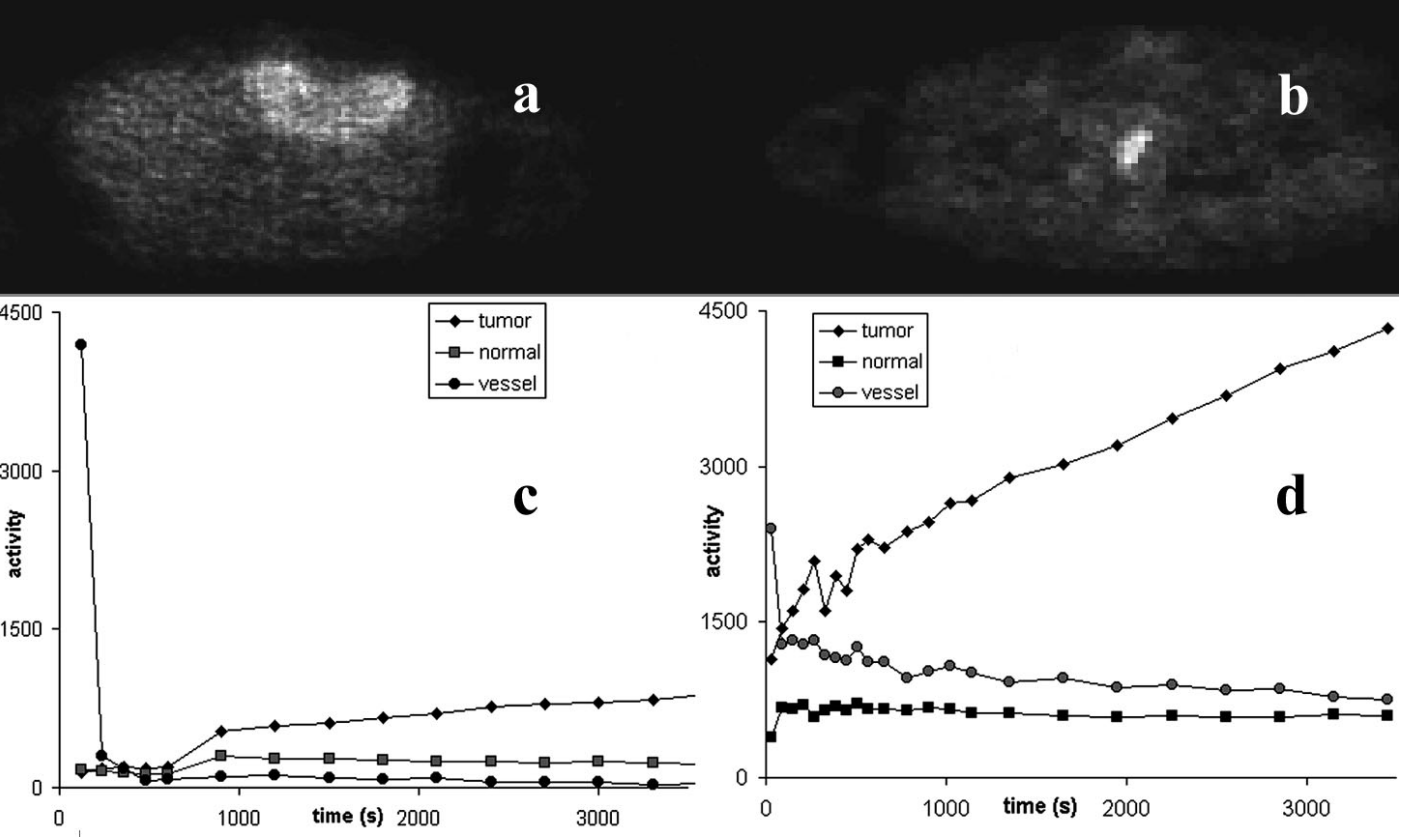
Clinical dynamic PET data showing: (a) a large lesion on the upper left liver lobe and (b) a colorectal tumor recurrence, and (c) and (d) the corresponding TACs.

Our study involved 17 patients with colorectal tumor recurrences, and one patient with liver metastasis, referred on the basis of clinical symptoms and radiological examinations. The final diagnosis was based on the histological data from surgical samples. Figure 2 shows images from the liver and one of the colon studies, corresponding to the late emission part of the dynamic data (i.e., the summation of the final four frames).

The dynamic PET studies were performed after intravenous injection of 300–370 MBq 18F-FDG for a period of 60 min. A 23-frame protocol was used (10 × 1 min, 5 × 2 min, and 8 × 5 min). The 18F-FDG was prepared according to Toorongian's protocol [40]. A dedicated PET system (ECAT EXACT HR+; Siemens, Erlangen, Germany) operating in the two-dimensional (2D)-mode (septa extended) was used for the patient studies. The system allows for the simultaneous acquisition of 63transverse slices with a theoretical slice thickness of 2.4 mm, and had an axial field of view of 15.3 cm. Transmission scans were obtained over a period of 10 min using three rotating germanium pin sources for the attenuation correction of the acquired emission images before injection of the FDG.

All the PET images were attenuation corrected, and an image matrix of 128 × 128 pixels was used. An iterative image reconstruction algorithm [41] was employed (weighted least-square method, ordered subsets, 4 subsets, and 6 iterations) and the standardized uptake values (SUV) were calculated as:

SUV=tissue concentration (MBq/g)injected activity (MBq)/body weight (g)
 MathType@MTEF@5@5@+=feaafiart1ev1aaatCvAUfKttLearuWrP9MDH5MBPbIqV92AaeXatLxBI9gBaebbnrfifHhDYfgasaacH8akY=wiFfYdH8Gipec8Eeeu0xXdbba9frFj0=OqFfea0dXdd9vqai=hGuQ8kuc9pgc9s8qqaq=dirpe0xb9q8qiLsFr0=vr0=vr0dc8meaabaqaciaacaGaaeqabaqabeGadaaakeaajaaqcqWGtbWucqWGvbqvcqWGwbGvcqGH9aqpkmaalaaajaaqbaGaeeiDaqNaeeyAaKMaee4CamNaee4CamNaeeyDauNaeeyzauMaeeiiaaIaee4yamMaee4Ba8MaeeOBa4Maee4yamMaeeyzauMaeeOBa4MaeeiDaqNaeeOCaiNaeeyyaeMaeeiDaqNaeeyAaKMaee4Ba8MaeeOBa4MaeeiiaaIaeeikaGIaeeyta0KaeeOqaiKaeeyCaeNaee4la8Iaee4zaCMaeeykaKcabaGaeeyAaKMaeeOBa4MaeeOAaOMaeeyzauMaee4yamMaeeiDaqNaeeyzauMaeeizaqMaeeiiaaIaeeyyaeMaee4yamMaeeiDaqNaeeyAaKMaeeODayNaeeyAaKMaeeiDaqNaeeyEaKNaeeiiaaIaeeikaGIaeeyta0KaeeOqaiKaeeyCaeNaeeykaKIaee4la8IaeeOyaiMaee4Ba8MaeeizaqMaeeyEaKNaeeiiaaIaee4DaCNaeeyzauMaeeyAaKMaee4zaCMaeeiAaGMaeeiDaqNaeeiiaaIaeeikaGIaee4zaCMaeeykaKcaaaaa@8502@

The SUV calculations were carried out using the last study frame (55–60 min, post injection). No partial volume correction was performed. However, SUV measurements were performed on volumes of interest spanning over several tomographic slices instead of using the conventional method averaging the measured concentration over an ROI drawn from a single slice.

## Results

### A. Application of PCA

**Figure 3:**
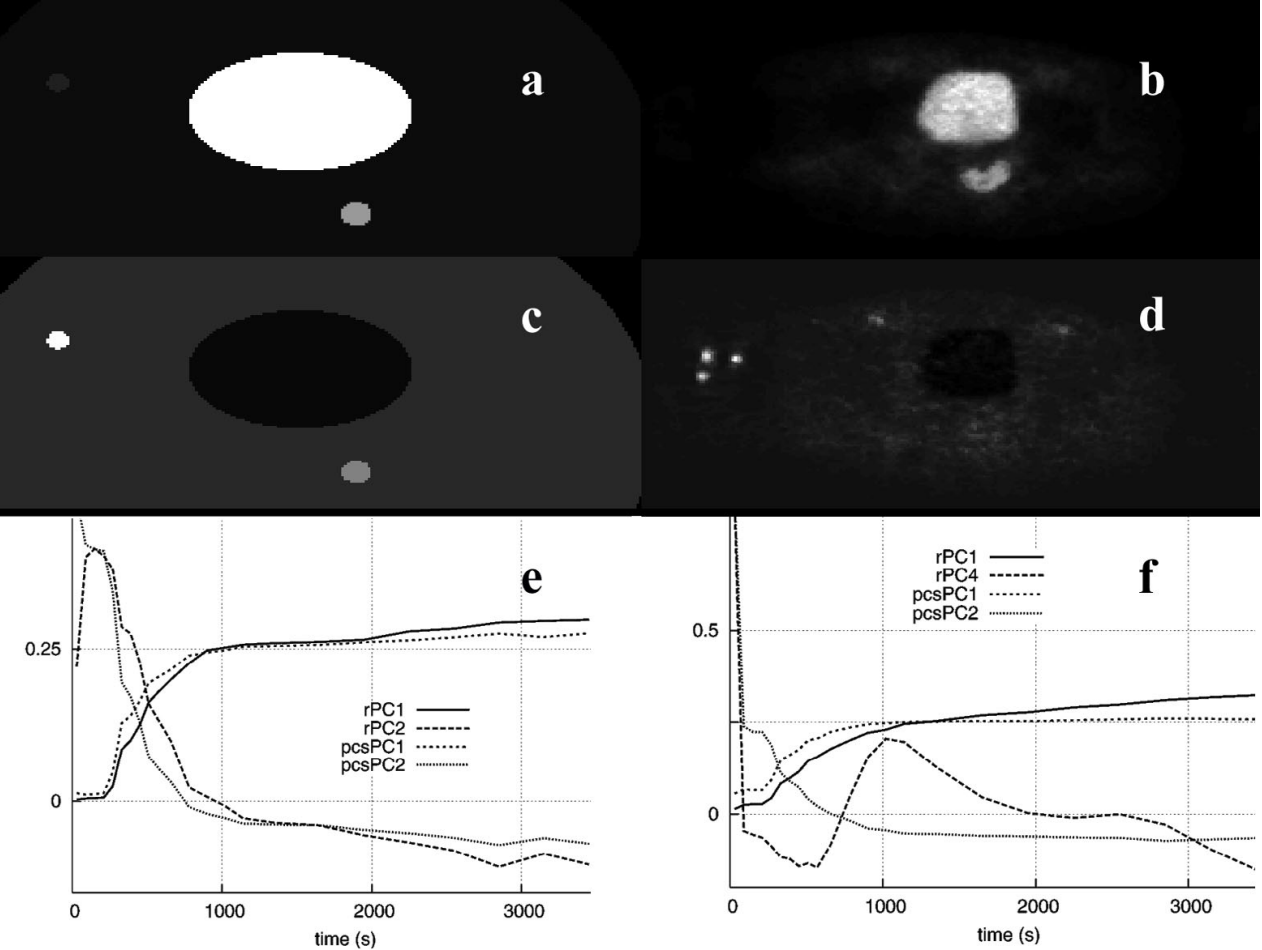
Image PCI1 (a) and image PCI2 (c) of the phantom study and the PCs (e) calculated using the raw data (rPC), and after applying the PCS preprocessing technique (pcsPC). Also shown are image PCI1 (b) and image PCI2 (d) of the corresponding clinical study calculated using the PCS preprocessed data and PCs (f) for both the raw and the PCS-transformed data.

Applying PCA to the synthetic data (figure 1) resulted in two PCIs (figure 3). In image PCI1 regions were depicted corresponding to the bladder and tumor of the phantom, whereas image PCI2 showed the blood vessel region in white and the tumor in dark gray. The PCS data transform did not change the images. However, the corresponding PC curves differed slightly. In both cases, curve PC1 resembled the bladder's TAC of the phantom data, which was in agreement with the structures present in the PCIs. However, transforming the raw data led to a shape of curve PC2 that was closer to the actual blood vessel's TAC.

When PCA was applied to the clinical study used to create the synthetic data (figure 1), the tumor in the vicinity of the bladder was clearly detected in image PCI1 (figure 3), whether the raw data (denoted as rPC) or PCS-transformed data (denoted as pcsPC) were used. The blood vessels were clearly shown in images PCI2 or PCI4, depending on whether or not the original data were preprocessed. In agreement with the results obtained using the phantom, the PC curves matched the measured TACs better using PCS-transformed data. Beyond the fourth principal component, the resulting principal component images contained mainly noise.

**Figure 4:**
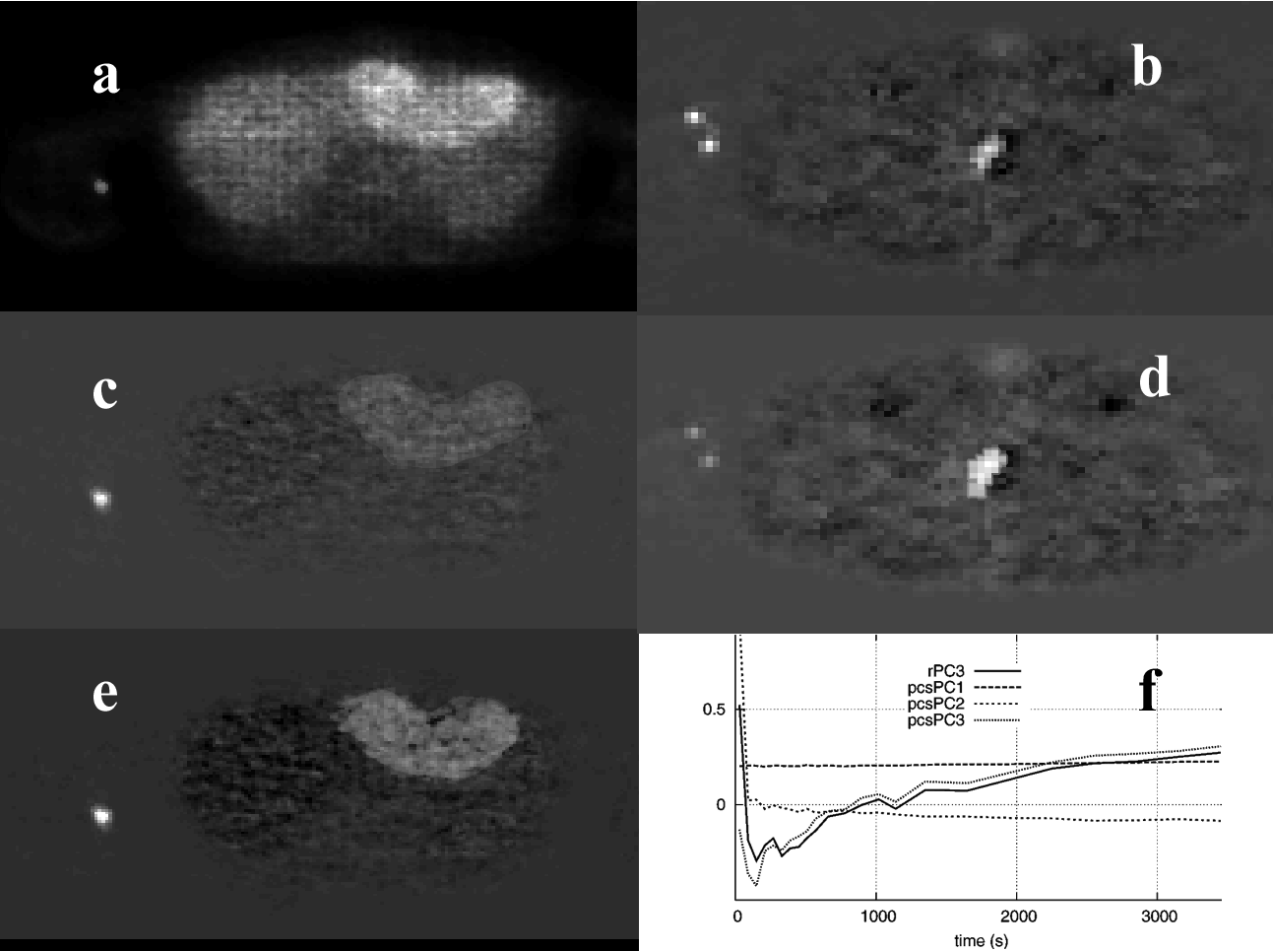
PC Images from the liver study: rPCI1 (a), rPCI3 (c), pcsPCI3 (e), and PCIs from the colon study: rPCI3 (b) and pcsPCI3 (d), with the corresponding PCs (f).

In the case of the liver study shown in figure 2, image PCI1 resembled a summed image of all the original image frames, where all the structures were visible (figure 4). Image PCI2 showed only the vascular components leaving the area covered by the lesion in black, whereas the third image (PCI3) contained a bright area corresponding to the lesion. The PCS transformation technique applied before PCA improved the lesion delineation in the corresponding PCI (PCI3).

Our results and the initial conclusions drawn were verified by applying the same analysis to the data obtained from 17colorectal tumor recurrence clinical studies. Only in image PCI3 were the lesions clearly visible in 14 of the cases, and in only in three cases did their small size, due to partial volume effects and possibly to physiologic activity in the surrounding tissues, not allow the direct correlation of image PCI3 to the tumor. Preprocessing the data resulted in PC images where the lesions were better delineated and the blood vessels could hardly be seen, which is in agreement with the corresponding PCs. Therefore, the PCA facilitated the detection and identification of structures in large dynamic FDG PET oncological studies.

### B. Application of ICA

**Figure 5:**
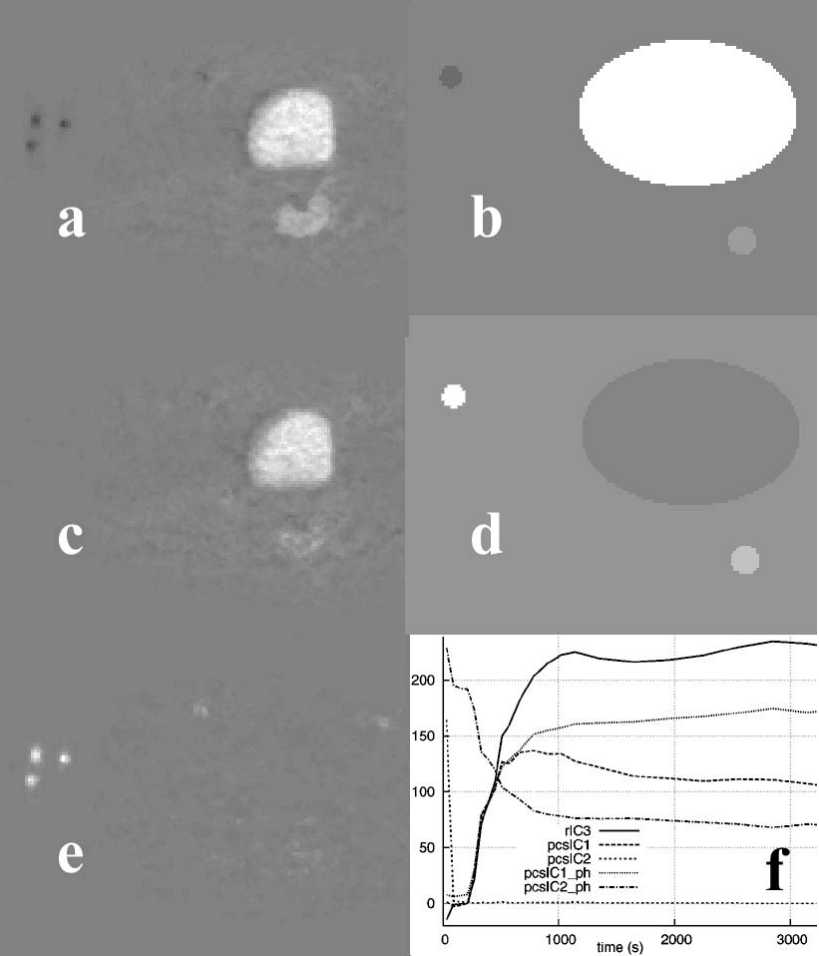
ICIs of the clinical study shown in figure 1 based on raw data rICI3 (a) and on PCS-transformed data pcsICI1 (c), pcsICI2 (e), and pcsICI1 (b) and pcsICI2 (d) for the phantom. The corresponding ICs for all the images are shown in (f).

Figure 5 shows the results from the application of a skew-sICA to the dynamic PET study shown in figure 1. When no preprocessing was applied to the data before SVD analysis, all the structures are present in the third raw Independent Component Image (rICI3), which was colored according to the kinetic characteristics (i.e., the bladder and tumor in white, and the blood vessels in black). On preprocessing the data, the bladder and blood vessels are shown correspondingly in the PCS-preprocessed ICI1 image (pcsICI1) and in pcsICI2, while the tumor was "guessed" in pcsICI1. The results are in agreement with the images obtained using the phantom data and the ICIs shown in figure 5 (right column).

**Figure 6:**
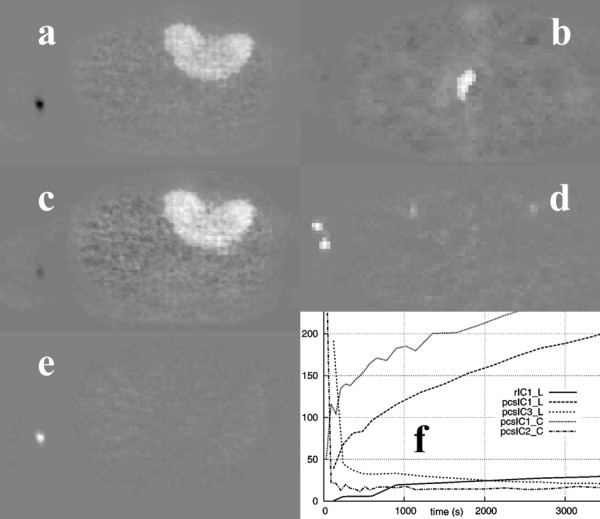
Images rICI1 (a), pcsICI1 (c), and pcsICI3 (e) from the liver study. Images pcsICI1 (b) and pcsICI2 (d) from the colon study. The corresponding ICs of the above images are shown in (f).

When applying the skew-sICA to the liver study, the lesion was displayed in image rICI1 in a bright color, and blood vessel was shown in a dark color, while in image rICI2, the blood vessel was clearly depicted as white, and the lesion could hardly be distinguished (figure 6). On transforming the data using the PCS, image pcsICI2 only displayed the blood vessel, whereas image pcsICI3 resembled image rICI1. However, the lesion in the latter could be distinguished using higher contrast.

In the case of the colorectal tumor recurrence clinical studies, the tumors are shown in bright and dark colors in images rICI1 and rICI2, respectively, while the blood vessels are displayed in bright colors in image rICI3. The PCS data transformations carried out before the ICA process produced similar results. The difference in activity levels between the lesion and normal tissue was higher in the colorectal tumors than in the liver metastasis (figure 2), and no transformation was required to improve the separation of the structures.

### C. Application of SM

**Figure 7:**
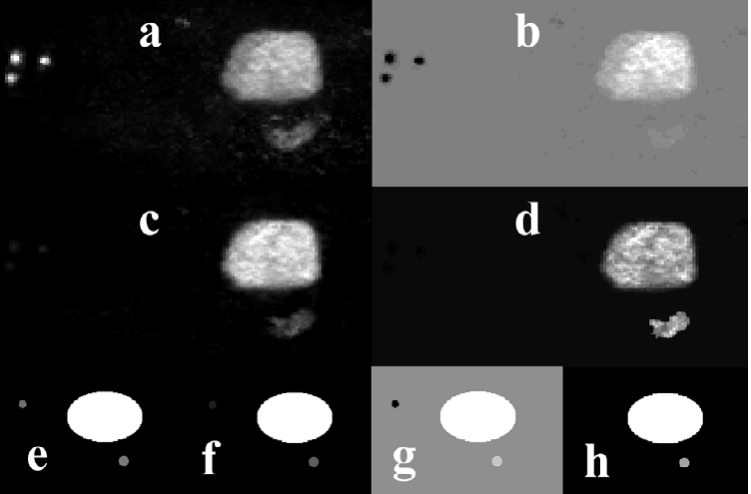
Similarity maps of the clinical PET study shown in figure 1 calculated using a reference ROI placed over the bladder, and using the SM measures of: SSQ (a), SQS (c), SC (b), and CS (d). Images (e-h) show the corresponding similarity maps for the simulated data of figure 1.

**Figure 8:**
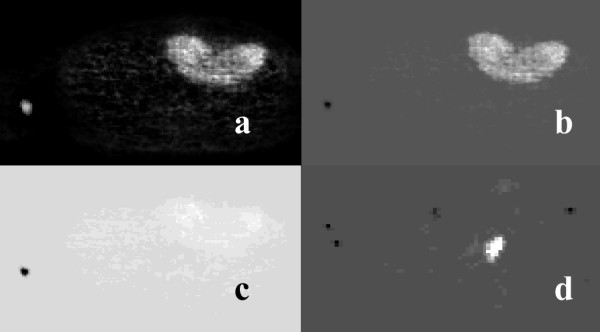
Similarity maps of the PET studies shown in Fig. 2 showing SQS (a), SC (c), and CS (b) from the liver study, and the CS (d) from the colon study. The reference ROIs were placed over the lesions in both cases.

Figure 7 shows similarity maps of the clinical and simulated studies shown in figure 1 that were calculated by placing an ROI over the bladder, and using the similarity coefficients SSQ, SQS, SC, and CS. Figure 8 shows similarity maps of the clinical studies shown in figure 2, calculated using a tumor rROI. In all cases, the similarity maps based on the COR and NCOR coefficients were very noisy, and it was difficult to separate different structures.

Tumors could be distinguished in the SM images using different levels of contrast and clarity. In the SSQ and SC maps, the lesions were difficult to detect, while use of the SQS and CS coefficients detected all the lesions (from the 21 individual lesions present in all the studies). However, in the SQS maps, both the blood vessels and the tumors had positive values, and are displayed in white. On the other hand, the CS coefficient provides a way of discriminating between these two different groups of structures, by assigning positive values to the tumors and negative values to the blood vessels, due to their different kinetic characteristics.

The contrast in the similarity images was measured using CR = (T-M)/M (where T and M are the mean activity distributions in the ROIs placed over the tumor and normal tissue mass areas, respectively). The resulting values ranged from 0.15 for the COR coefficient (showing the lesions were at a similar contrast level to that of normal tissue) and 2.46 for the NCOR coefficient, to 54 for the CS metric. The SUV images exhibited values around 34.

## Discussion

In addition to the traditional diagnostic procedure based on the visual inspection of tomographic images, semiquantitative measures based on the SUV normalization of tracer concentrations of the injected activity and body weight are becoming common in the clinical praxis of oncological PET studies [42]. It should be noted that SUV-based evaluation requires a well-calibrated PET platform to produce those semi-quantitative results.

SUV-based evaluation can also be used to characterize the later stages of glucose uptake of tissues by ignoring the kinetics of this predominantly dynamic process, which may be able to provide valuable information on the molecular events that characterize tumor development and associated vasculature, as well as its specific resistance to treatment. The use of SUV as a classification method for tissue areas as being either benign or malignant is still being discussed by nuclear medicine physicians and oncologists [43,44], and depending on the conditions under which the study has been performed and the data have been preprocessed, the use of SUV can be misleading in PET studies [45]. PCA automatically generates images that correlate with the activity of different structures present in a dynamic PET study, facilitating visual inspection and the application of compartmental analysis, since it provides a tool for a more accurate selection of ROIs in lesions and/or blood vessels to allow for further parametric analysis of the dynamic sequences.

ICA has also been evaluated to see if this approach can further improve on the results obtained so far. Being data-driven methods, both PCA and ICA imply that a particular statistical model is used, whether or not this model is made explicit. The model implicit in PCA is that different modes are Gaussian and uncorrelated, whereas the ICA model is that different modes are non-Gaussian and independent. Therefore, ICA with proper preprocessing is expected both to decorrelate the signals and to reduce any higher order statistical dependency and the contribution due to the source that corresponds to the noise. Using this approach, structures in an image series can be separated easily without the need for precise a priori anatomical information.

A dynamic PET image sequence represents sample measurements of the FDG distribution with time, as this is described using an underlying compartmental model [2]. The images are generated using the assumption that the image frames are a linear combination of spatially independent images, in our case, tumors, blood vessels, bladder, normal tissue, and noise. It should be noted that the spatial independence does not interfere with the fact that the TACs are correlated in time according to the FDG compartmental model. Since the source components are non-Gaussian in a general sense, and are considered spatially independent, the ICA approach is assumed to be the most appropriate method for performing blind source separation in dynamic FDG PET image sequences.

The results discussed above show that the skew-sICA approach automatically generates images where structures with different kinetic characteristics, such as tumors and blood vessels, can be readily discriminated, since they are assigned opposite values. The possibility of performing quantitative analysis of dynamic PET studies using the skew-sICA approach, and the assessment of the performance of spatiotemporal ICA experiments are presently under investigation.

Two previously described similarity measures were used to calculate the similarity maps: COR and NCOR, and four new similarity measures were introduced: SSQ, SQS, SC, and CS. The use of these correlation-based similarity metrics was selected as being the most commonly used methodology used in comparisons of the similarity between images or image segments. SM depicts all the structures present in the dynamic studies in a single image. The generation of similarity maps is not automatic, as in the case of PCA, since these maps represent the contrast of a lesion area versus muscle tissue, after the placement of an ROI over the lesion and the blood vessels, respectively.

The application of COR and NCOR maps can be used to discriminate the structures present in the dynamic phantom data set. However, these were found to be ineffective in separating structures in clinical data. The new similarity coefficients proposed here in equations (5)–(8) revealed the structures of interest on visual inspection. In particular, CS, as defined in equation (8), provided better parametric images, and could be the method of choice as far as discriminating between a tumor and other structures is concerned, both from simulated phantom studies and clinical data from PET studies of colorectal tumor recurrences. In its formulation, CS basically resembles NCOR, defined in equation (4). However, the numerator in CS is raised to the third order power, which helps to increase the contrast in CS-calculated similarity maps for low counts and high-noise PET images, and it also includes a calibration parameter in the denominator. This contrast-enhancement property of the CS approach is less pronounced in the SQS approach of equation (7), as the square power is used instead, and this limits the value range for this similarity criterion.

Parametric images calculated using each of the techniques discussed depict structures that share the same kinetic characteristics. However, they do not provide quantitative information. These images may contain negative values, corresponding to pixels within the TACs of different kinetic characteristics, and are finally normalized for display, which facilitates the discrimination of the regions of interest.

The PCs and ICs generated by the PCA and ICA approaches may also contain negative values in the time domain, even though they do not coincide with physiologically meaningful TACs. Their shape (either increasing or decreasing with time) rather than their absolute values agrees with the type of TACs expected according to the structures present in the corresponding images. Therefore, they can be used for the identification of regions of interest.

Depending on the type of structures present in each dynamic study, the difference in activity levels among them, and the method employed in their analysis, preprocessing the original data could improve lesion delineation, and possibly its detectability too, as shown when PCS was applied prior to PCA in the liver and colorectal studies, or prior to ICA in the liver study. In all these studies, the shape of the PCs of the PCS-transformed data was much closer to the shape of the real, measured TACs of similar structures. In some cases, the application of preprocessing had no visible effect on the resulting images, e.g., when ICA was applied to the colorectal PET studies, or it could even hinder the detection of lesions, as shown in figure 5.

All the methods described require that the image frames for the same tomographic slice be spatially registered. These images need to be checked for spatial registration to correctly classify voxels or the volumes/regions of interest based on similarity criteria. Therefore, patient motion and respiratory artifacts should be corrected prior to the application of these methods to dynamic PET images.

## Concluding remarks

The PCA, ICA, and SM techniques represent efficient methods for data reduction of large PET dynamic image sequences. They support visual interpretation of dynamic studies and can assist the application of compartment modeling. The methods developed here represent promising alternative techniques for the fast, independent, quantification of any kinetic model, and this is useful when the retrieval of the input function is complicated. Therefore, the treatment planning and assessment of angiogenesis-blocking drugs using PCA, ICA, and SM can now be investigated. In the case of SM processing, manual selection of the reference ROI can be time consuming and prone to operator bias, and therefore further research is required for the development of a semiautomatic technique for the optimum selection of a reference ROI. The methods discussed permit the study of the temporal behavior of dynamic PET image sequences, and allow for the extraction of valuable information in real time to assist the physician in obtaining a diagnostic decision, which may be difficult under other circumstances.
